# Supplementary material for: Real-time visualization of phagosomal pH manipulation by Cryptococcus neoformans in an immune signal-dependent way
Source: Front Cell Infect Microbiol. 2022 Sep 23;12:967486. doi: 10.3389/fcimb.2022.967486 (PMC9538179; doi:10.3389/fcimb.2022.967486)
Supplement: Supplementary file 1 [file DataSheet_1.pdf]

Text S1. Supplementary Methods

1. **Strains, cell lines, and growth conditions**. Strain used were *C. neoformans* serotype A strain KN99α expressing cytoplasmic mCherry, the yeast-locked *C. albicans cph1/cph1 efg1/efg1* double mutant strain, and the *S. cerevisiae* strain BY4741 expressing cytoplasmic mCherry. All fungal strains were maintained at -80 °C and grown at 30 °C on yeast-peptone-dextrose (YPD) plates. Overnight cultures were grown in 5 mL of YPD at 30 °C with shaking (230 rpm). The human monocytic cell line THP-1 (ATCC TIB-202) was grown in THP-1 complete media (RPMI-1640 with L-glutamine supplemented with 1 mM sodium pyruvate, 0.05% β-mercaptoethanol, 10% FBS, and 1X Pen-Strep solution (100 units/mL penicillin and 100 μg/ml streptomycin)) and passed 2 – 3 times per week. Every month a new low-passage stock was thawed and the cells were never used after 12 passages. For differentiation into macrophages, the THP-1 cells were treated with 0.16 mM phorbol 12-myristate 12-acetate (PMA, Millipore Sigma) for 48 hr with a subsequent recovery step without PMA for 24 hr. Prior to using the macrophages on cellular or infection assays, their physical characteristics were assessed visually: flatten, attached cells, with dense granules.
2. **Labeling of fungal cells with pHrodo-Green**. Stock solutions of pHrodo iFL Green (P36012, Thermo Fisher Scientific) were prepared in DMSO according to the manufacturer's instructions (10 mg/mL) and stored at -20°C in single use aliquots (5 μL). A fresh aliquot was thawed for each experiment. Fungal cultures grown overnight at 30°C with shaking (225rpm) were diluted to an OD_{600} of 0.2 and grown for two doublings. The log phase cultures were washed in PBS and 1 x 10^8 cells opsonized with 1 mL of 40% human serum for 30 min at 37°C with rotation. Serum was obtained from healthy donors with informed consent under a protocol approved by the University of Notre Dame Institutional Review Board. We opsonized first to avoid having the complement protein modified with the pHrodo dye, although it is uncertain if that will have an effect on phagocytosis. The opsonized fungal cells were spun down and resuspended in 100 μL of 100mM sodium bicarbonate (freshly prepared from a 1M stock). Cells are mixed well before adding 5 μL of the pHrodo Green stock to a final concentration of 500 μg/mL and incubated in the dark for 30 min at 37°C with rotation. Cells were washed twice with PBS and resuspended in 1 mL of imaging media (DMEM without phenol red and L-glutamine, supplemented with 10% FBS, 100 units/mL Penicillin- 100 μg/mL Streptomycin solution, 2.5 mM L-glutamine, and 10mM HEPES, pH 7.0). At this point, depending on the target MOI or amount of fungal cells needed, an aliquot was transferred into a new tube with imaging media to obtain a concentration of 10^7 cells/mL. To test the efficiency of the staining, 200 μL can be removed and split into two tubes. These can be spun down and one tube resuspended in PBS while the other in acetate buffer. You can use these tubes to determine optimal exposure times. To the remainder solution, the cytokines IFNγ or IL-4 are added when necessary.

   Other adjustments: when using non-fluorescent fungi, the pHrodo staining was carried out in PBS such that CFW could be added at the same time. For certain cryptococcal mutants, sonication in a water bath was necessary to avoid clumping of cells during staining procedures.
3. **Time Lapse Microscopy**. Coincubation of opsonized, pHrodo-stained cells with differentiated THP-1 cells was done in 35mm glass-bottom dishes (MatTek Corporation). The dishes were

seeded with $5x10^5$ THP-1 cells by resuspending the cells in 500 μL of differentiation media (THP-1 complete media with 0.16mM PMA) and pipetting onto the coverslip in the center of the dish. This was incubated 1 hr at 37°C/5% CO_2 before adding 2.5 mL of prewarmed differentiation media to the dish. This process results in a density of $1.67x10^5$ cells/mL and ensure that most THP-1 cells are attached on the glass coverslip rather than floating or weakly attached to the plastic around the coverslip on the dish. Following the 48 hr incubation with differentiation media, the dishes are washed with prewarmed DPBS and media replaced with complete media without PMA for a 24 hr recovery step. If needed, during the recovery step cytokines were added for polarization of the macrophages. For M1 macrophages, the media contained 200 ng/mL of recombinant human IFNγ (R&D Systems). For M2 macrophages, the media contained 50 ng/mL of recombinant human IL-4 (Shenandoah Biotechnology). Prior to imaging, culture media was replaced with prewarmed imaging media containing appropriate cytokines and opsonized and pHrodo-stained *C. neoformans*, *C. albicans*, or *S. cerevisiae* at a MOI of 5, 1, or 1, respectively. The dish was immediately transferred to a PeCon (Erbach, Germany) P S compact stage-top incubator preheated to 37 °C with 5% CO_2. A Zeiss Axio Observer 7, with an Axiocam 506 mono camera and a 40x objective was used to image 2x2 fields of view every 3 minutes for at least 180 minutes.

4. **Flow Cytometry**. 900,000 THP-1 cells were seeded and PMA-differentiated per well in a 6-well plate as described above. Opsonized and pHrodo-stained *C. neoformans* or *S. cerevisiae*, as described above, were added to each well. A T25 flask of THP-1 cells was also seeded together with a culture of unstained fungi to provide negative controls for the flow cytometry analysis. Other controls included pHrodo-stained cells in acetate buffer, and mCherry-expressing fungi. At every timepoint (30, 60, and 120 min), the wells were washed 3X with DPBS to remove most of the extracellular and weakly adherent fungal cells, and the macrophages were lifted with TrypLE without phenol red (Thermo Fisher Scientific). The cells were collected and resuspended in cold DPBS and immediately analyzed by flow cytometry (BD LSRFortessa). The gating strategy is described in Fig. S3.
