# Supplementary material for: Real-time visualization of phagosomal pH manipulation by Cryptococcus neoformans in an immune signal-dependent way
Source: Front Cell Infect Microbiol. 2022 Sep 23;12:967486. doi: 10.3389/fcimb.2022.967486 (PMC9538179; doi:10.3389/fcimb.2022.967486)
Supplement: Supplementary file 2 [file Table_1.docx]

**Table S1**. Kinetics of acidification (in minutes).

| Infected with: | *S. cerevisiae* | | | | *C. neoformans* | | | | *C. albicans*^a^ | | | |
| --- | --- | --- | --- | --- | --- | --- | --- | --- | --- | --- | --- | --- |
|  | M0 | M1 | M2 | Overall | M0 | M1 | M2 | Overall | M0 | M1 | M2 | Overall |
| Time to fluorescence after uptake (behavior 1) | 19.7±  10.5 | 14.3± 9.3 | 20.3± 16.2 | 16.9± 12.5 | 17.6±  9.1 | 18.4± 9.9 | 24.3± 13.3 | 22.8± 12.7 | 20.6± 13.7 | 14.1± 6.4 | 25.25± 16.3 | 21.3± 14.4 |
| Time to fluorescence after uptake (behavior 2) | 21^b^ | 21.6± 13 | 27 ^b^ | 22± 12.7 | 28.3± 12.9 | 25± 25.1 | 34.4± 12.6 | 25.3± 13.9 | 25.5± 6.4 | 11± 3.5 | 36 ^b^ | 20± 11.2 |
| Both behaviors combined | 19.9± 10.4 | 15.8± 12.7 | 20.5± 16.1 |  | 20.5± 11.1 | 19.4± 12.6 | 25.9± 13.6 |  | 20.8± 13.5 | 13.8± 6.2 | 25.4± 16.2 |  |

^a^ SC5314 *cph1*/*cph1* *efg1*/*efg1* strain is a hypha-deficient mutant

^b^ Only one instance was recorded.
