# Supplementary figures and images for: Real-time visualization of phagosomal pH manipulation by Cryptococcus neoformans in an immune signal-dependent way
Source: Front Cell Infect Microbiol. 2022 Sep 23;12:967486. doi: 10.3389/fcimb.2022.967486 (PMC9538179; doi:10.3389/fcimb.2022.967486)

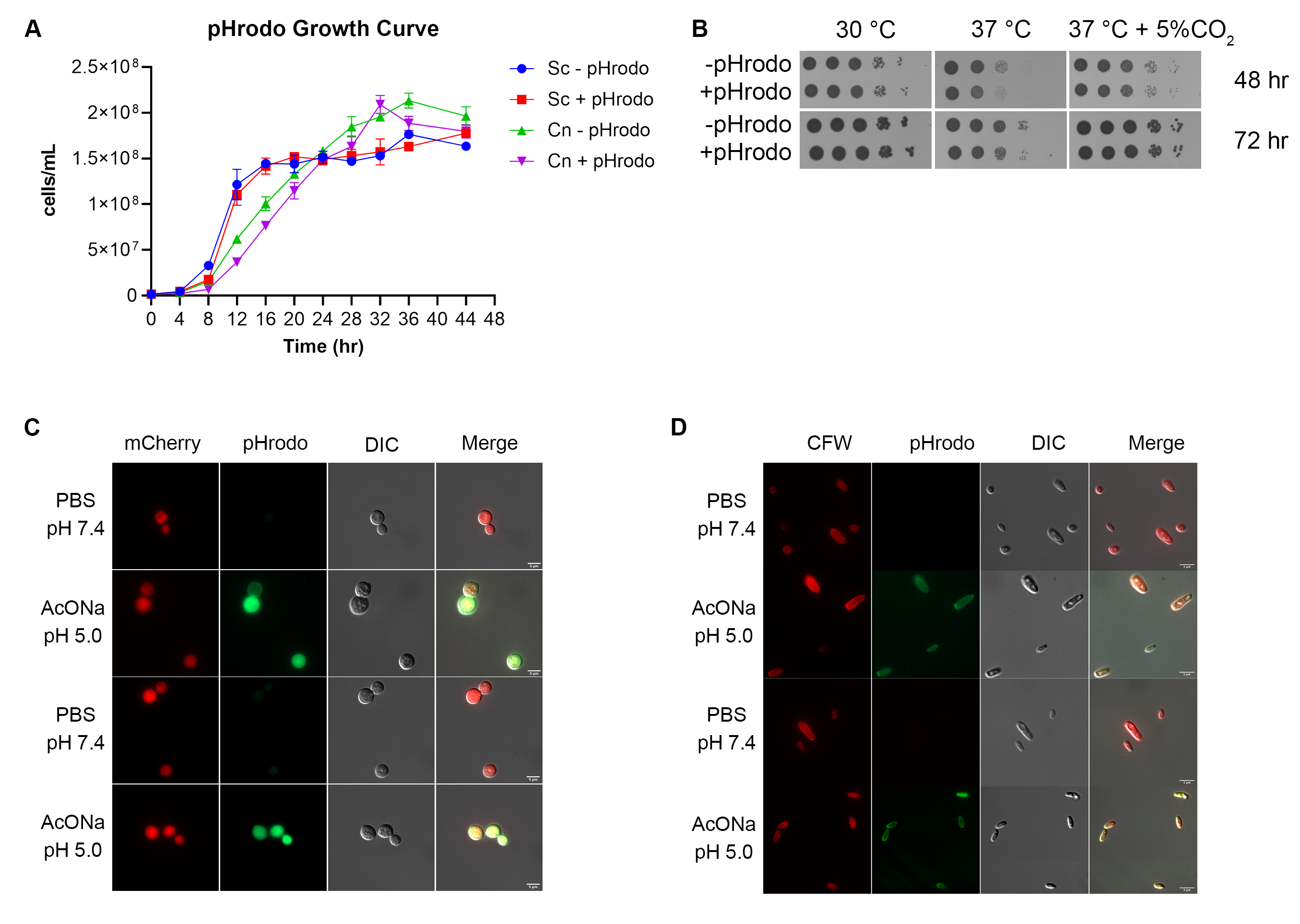

Supplement: Supplementary Figure 1 — Characteristics of pHrodo-stained fungal cells. (A) Growth curve of unstained and pHrodo-stained S. cerevisiae (Sc) and C. neoformans (Cn) in YPD media at 30°C over 48 hr. No effect of pHrodo staining is seen in either of the fungi. (B) Dot spot growth analysis in RPMI-agar plates of Cn under the indicated conditions. Images were taken at 48 and 72 hr. (C, D) Representative images of pHrodo-stained (C) Cn and (D) yeast-locked C. albicans (Ca) cells after sequential resuspension in PBS, pH 7.4, and sodium acetate buffer (AcONa), pH 5.0, showing that the pHrodo staining is responsive to changes in pH. Ca cells are not fluorescent and were counterstained with CFW. The CFW fluorescence was artificially colored red for ease of view. Scale bars represents 5 μm. [file Image_1.tif]

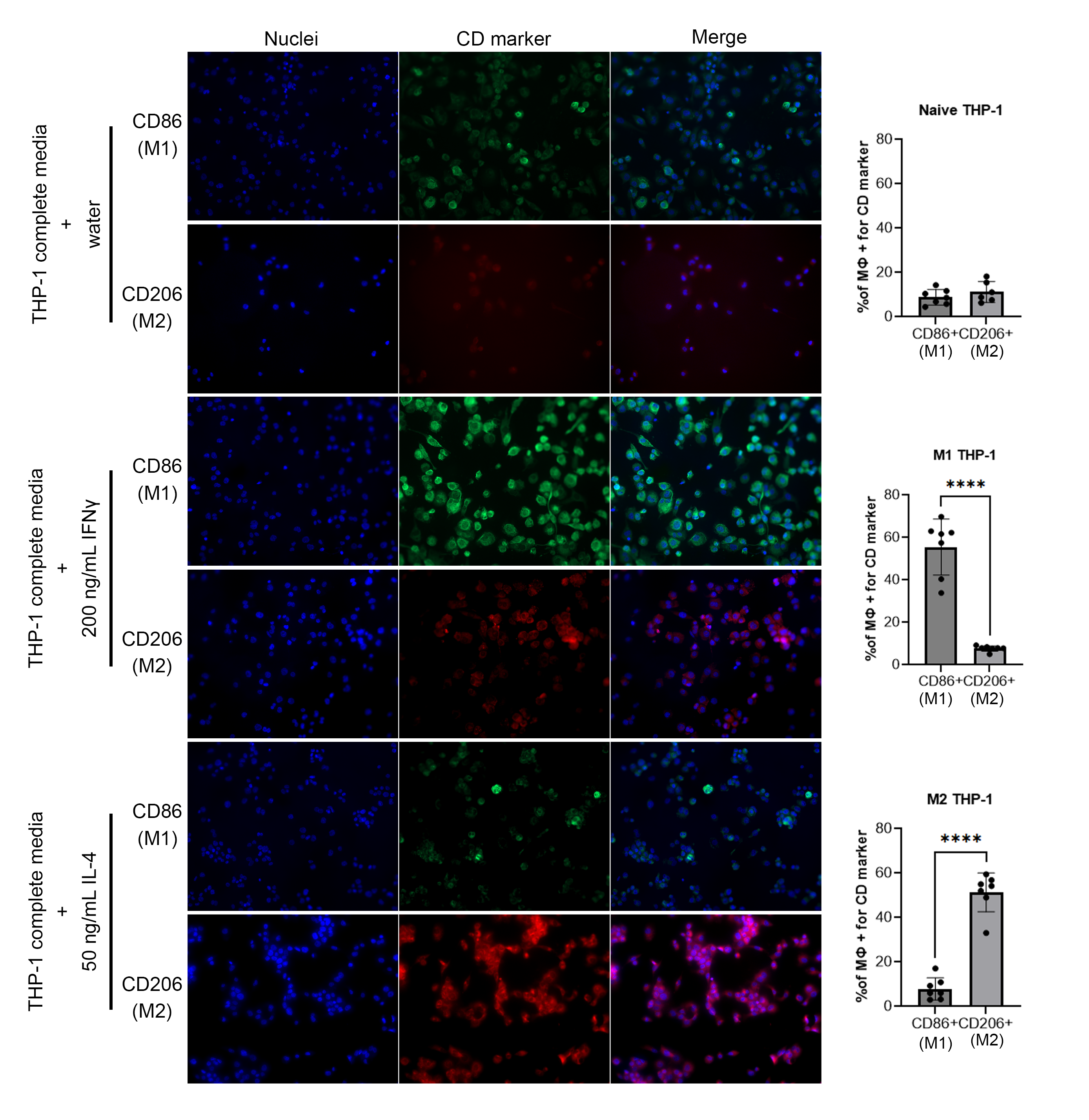

Supplement: Supplementary Figure 2 — Polarization of THP-1 cells using IFNγ or IL-4 treatment. THP-1 cells were treated with IFNγ, IL-4, or vehicle (water) as described in the Methods section. Shown are representative fields of views of immunofluorescence analysis using DAPI (blue) to stain nuclei; anti-CD86 antibody (artificially colored green); and anti-CD206 antibody (artificially colored red). The percentage of cells positive for each marker is quantified on the right. Each circle represents a coverslip, 2 coverslips per biological independent experiment. Statistics are unpaired t-tests comparing the two conditions. ****, P < 0.0001. [file Image_2.tif]

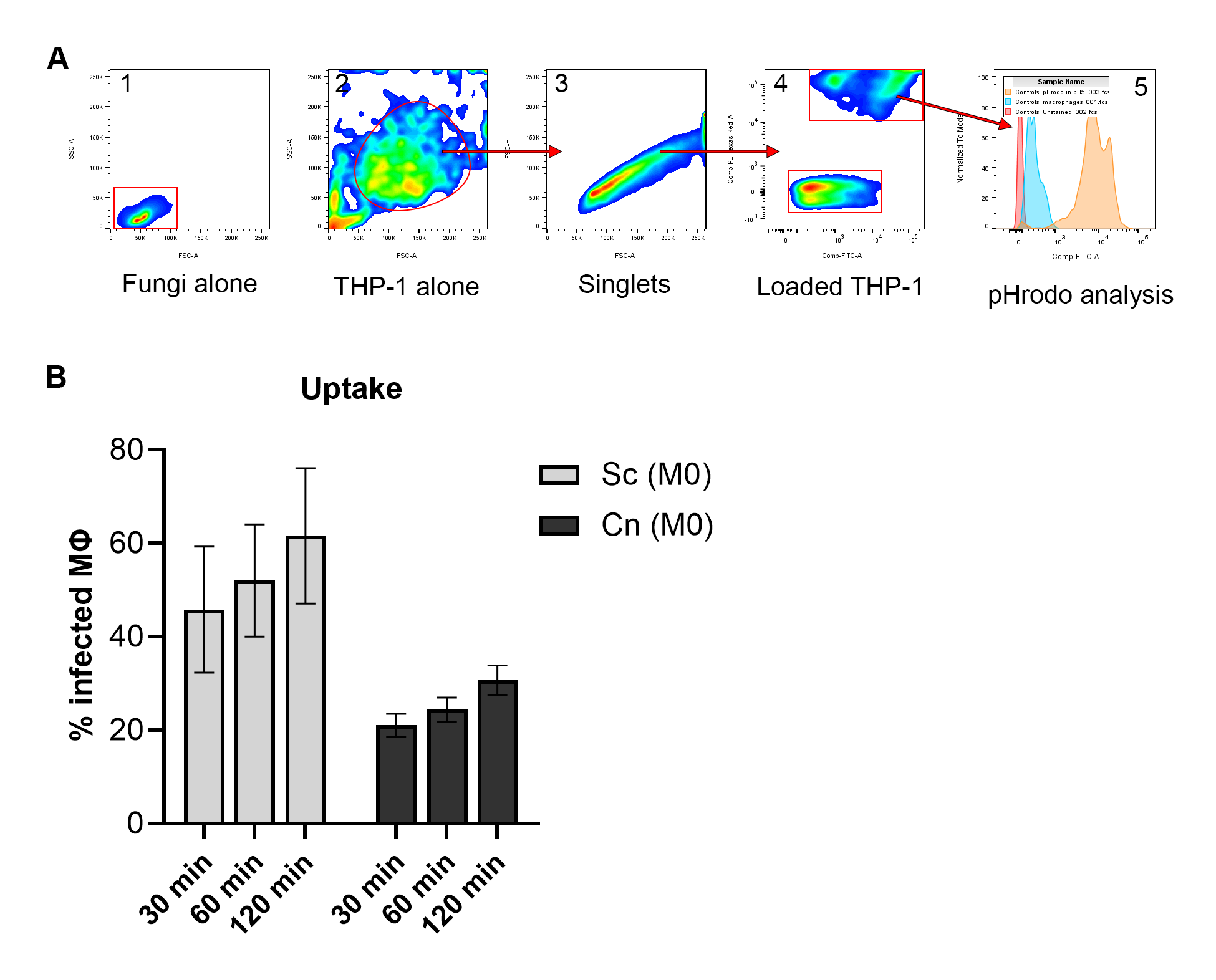

Supplement: Supplementary Figure 3 — Flow cytometry analysis. (A) Representative plots showing the flow cytometry gating strategy. First, unstained fungal and host cells are analyzed to determine their position in the size-scatter plot. These positions are used to eliminate free fungi (and cell debris) and select healthy THP-1 cells (gates 1 and 2). The healthy THP-1 cell population is used to select for single cells (gate 3). The singlets are then used to select for the population positive in mCherry (gate 4). The population negative to mCherry are uninfected THP-1 cells. Lastly, the infected population is used to analyze pHrodo signal (gate 5). (B) Uptake of fungal cells from a representative flow experiment using M0 macrophages. Despite using only an MOI of 1 (versus an MOI of 5 for C. neoformans), S. cerevisiae (Sc) is phagocytosed more avidly than C. neoformans (Cn). This data comes from Gate 4, the mCherry-positive population represents infected macrophages. The values in here were calculated by dividing gate 4 by gate 3. [file Image_3.tif]

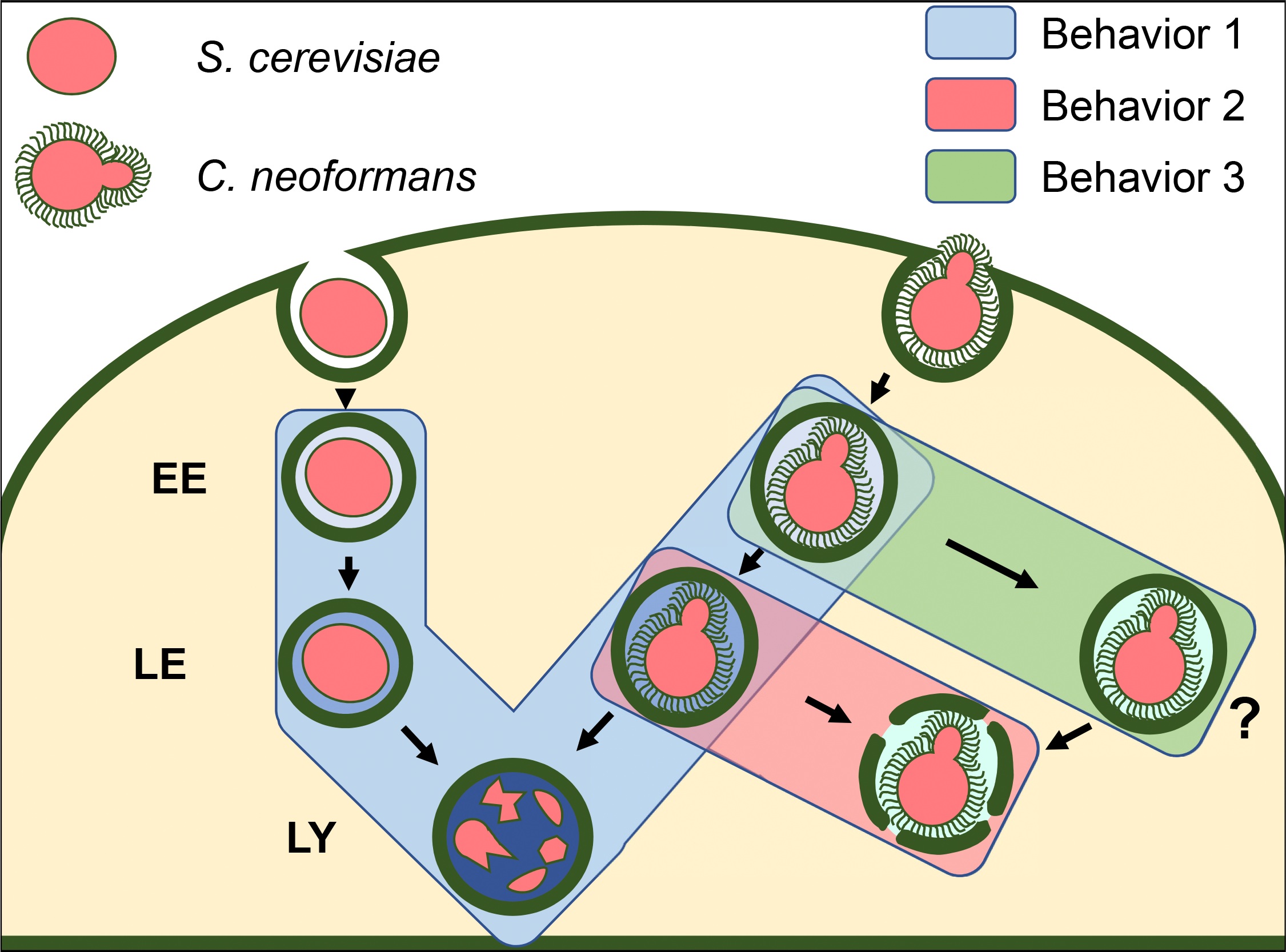

Supplement: Supplementary Figure 4 — Model summarizing our findings. We found three behaviors in cells infected with either S. cerevisiae (unbudded pink cell) or C. neoformans (budded pink cell surrounded by capsule). Behavior 1 (blue path) is shared between the two fungi and presumable results in formation of a fully-functional phagolysosome, where the fungal cells are destroyed. Behavior 2 (red path) deviates from the normal path and results in loss of acidification. This can happen in part by phagosomal membrane permeabilization (broken dark green outline). Behavior 3 (green path) results in cells that never acidify, hence this path represents an unknown phagosomal compartment (depicted by ‘?’). This compartment never acidifies so it could also result in phagosomal permeabilization, but we would not be able to see it in our live imaging. [file Image_4.tif]
